# Bisphenol A disrupts mitochondrial homeostasis and promotes metabolic alterations in cervical cancer cells

**DOI:** 10.1007/s11356-026-38082-3

**Published:** 2026-07-30

**Authors:** Nadeem Ghani Khan, Divya Adiga, Kannath U. Sanjay, Padmalatha Satwadi Rai, Shama Prasada Kabekkodu

**Affiliations:** 1 Department of Cell and Molecular Biology, Manipal School of Life Sciences, Manipal Academy of Higher Education, Manipal, Karnataka India; 2 Department of Biotechnology, Manipal School of Life Sciences, Manipal Academy of Higher Education, Manipal, Karnataka India

**Keywords:** Bisphenol A, Mitochondrial dysfunction, Cervical cancer, Oxidative stress, Metabolic reprogramming, Molecular docking

## Abstract

Bisphenol A (BPA), a synthetic endocrine-disrupting chemical, has been implicated in several diseases, including cancer. However, its effects on mitochondrial function in cervical cancer (CC) remain poorly understood. We investigated the effects of BPA on mitochondrial structure and function in SiHa and CaSki CC cell lines. Confocal microscopy revealed that BPA (1 nM–1 µM) disrupted mitochondrial morphology, increased circularity (^*^p < 0.05), and reduced branch length and content. BPA increased mitochondrial ROS (mtROS) and Ca^2^⁺ levels, decreased mitochondrial membrane potential (MMP) and mitochondrial mass, induced mtDNA depletion, and impaired oxidative respiration (OCR). Notably, BPA increased glucose uptake and lipid peroxidation while reducing the antioxidant (GSH and CAT) activity. A dose-dependent increase in mitochondrial lipid droplets (*p* < 0.001) was accompanied by opposite ATP trends: ATP levels increased in SiHa cells but decreased in CaSki cells. Western blot and qRT‒PCR revealed the cell type–specific regulation of PGC1α, TFAM, and DRP1. Molecular docking revealed strong binding of BPA to IDH2 (docking score − 7.38), MAOB (− 6.29), and HK2 (− 3.208), which was further validated by stable MD simulation. Thus, BPA may induce mitochondrial dysfunction, metabolic changes, and oxidative stress in CC cells in vitro, suggesting a potential role in cervical carcinogenesis that warrants further in vivo investigation.

**Graphical abstract:**

**Figure:**
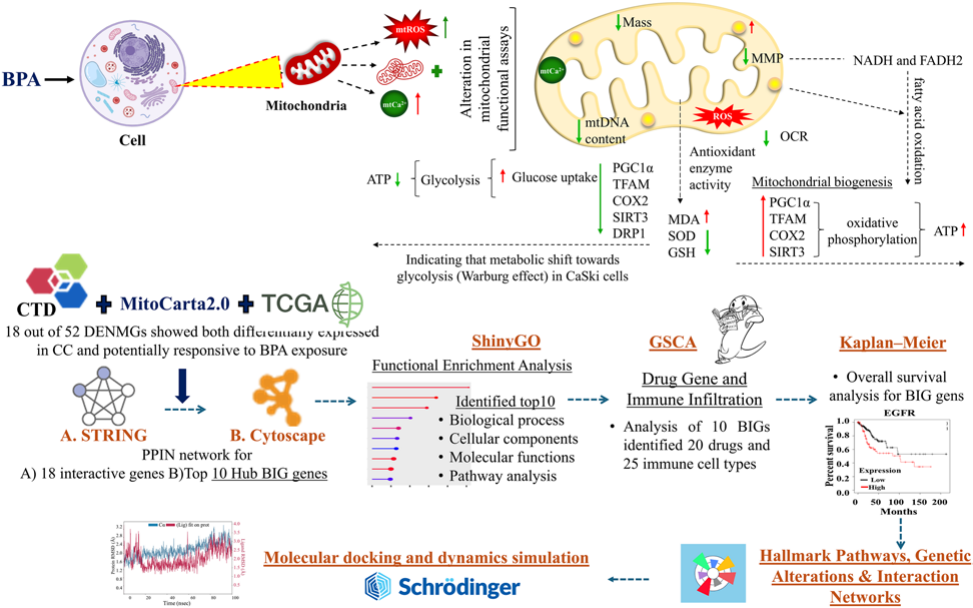

**Supplementary information:**

The online version contains supplementary material available at 10.1007/s11356-026-38082-3.

## Introduction

BPA is a well-characterized endocrine-disrupting chemical applied as a plasticizer to improve the physicochemical properties of plastics (Hafezi and Abdel-Rahman 2019) . Humans are continuously exposed to BPA through its leaching from consumer products (Vandenberg et al. 2007). It has been identified in the body fluids (amniotic fluid and urine), placental tissue, and the reproductive system. BPA has been linked to a range of human diseases (Aris 2014; Cantonwine et al. 2013). The structural similarity between BPA and estradiol enables it to bind to both G protein-coupled estrogen receptors (GPERs) and estrogen receptors (ERs) and thereby facilitating the expression of downstream target genes (Yuan et al. 2023). Several cell and tissue types exposed to BPA showed oxidative stress, DNA damage, and epigenetic modifications (Gassman 2017). Emerging evidence has proposed that BPA exposure might function as a potential risk for prostate and breast cancers. Nonetheless, further extensive research is necessary to determine the link between BPA exposure and the risk of cancer (Prueitt et al. 2023; Salamanca-Fernandez et al. 2021; Zhou et al. 2025). Although BPA is not classified as a human carcinogen, many studies recommend classifying it as a “Group 2A” carcinogen (Seachrist et al. 2016).

Cervical cancer (CC) is the predominant cancer impacting women residing in developing and underdeveloped countries (Catarino et al. 2015). CC pathogenesis is primarily driven by viral oncoproteins produced by high-risk HPV strains (Onwuamah et al. 2023). Interestingly, only a subset of high-risk HPV-infected women develop CC, suggesting the role of epigenetic factors and environmental factors in disease pathogenesis (Da Silva et al. 2021). The relationship between BPA exposure and CC or its precursor lesions remains underexplored in the scientific literature (Khan et al. 2024; Ma et al. 2015). By activating the ERK/AKT pathway and inducing MMP-2/9 expression, BPA treatment enhances the malignant behaviors of CC cells in culture, including their proliferative, migratory, and invasive capacities (Ma et al. 2015). BPA treatment promoted more aggressive phenotypes in both SiHa and CaSki cell lines (Khan et al. 2024; Ma et al. 2015). BPA-treated CC cell lines showed increased proliferation, migration, and invasion with simultaneous increase in total ROS, Ca^2+^, and lipid droplet levels (Khan et al. 2024). BPA triggered proteins of epithelial–mesenchymal transition (EMT) pathways and Wnt pathway. An *in silico* analysis of BPA-exposed gene expression datasets revealed overlapping cancer-promoting genes shared between BPA-responsive signatures and CC (Khan et al. 2024). While these phenotypic changes were documented, the underlying mechanisms, particularly the role of mitochondrial dysfunction, remained unexplored. Therefore, we aimed to systematically evaluate BPA-induced mitochondrial changes in CC using CC cell lines.

Mitochondrial dysfunction is recognized as a key cellular alteration in CC development (Srinivasan et al. 2017; Yang et al. 2025). Earlier studies have shown BPA to induce mitochondrial structure and functions (Nayak et al. 2022). Mitochondrial alterations caused by BPA include changes in mitochondrial structure, impaired energetics, dysfunction of ETC complexes, and increased oxidative stress (Nayak et al. 2022). While mitochondrial dysfunction is recognized as a key mechanism of BPA toxicity, its specific role in CC remains unexplored. Therefore, we systematically evaluated BPA-induced mitochondrial perturbations in CC cell line models. This study provides novel in vitro evidence linking BPA-driven mitochondrial dysfunction to potential mechanisms relevant to cervical carcinogenesis.

## Materials and methods

### Cell lines and BPA treatment

We used SiHa and CaSki cells that are authenticated by STR profiling. Experiments were conducted in DMEM and RPMI medium supplemented with 10% FBS for SiHa and CaSki cells, respectively. BPA treatment was performed under serum-free conditions at two concentrations (1 nM and 1 μM) for specified durations to assess the dose- and time-dependent effects. The control groups consisted of cells treated with dimethyl sulfoxide (DMSO). Medium without Phenol Red served as the basal medium for all experimental treatments (Khan et al. 2024). Imaging experiments were conducted using DMi8-TCS-SP8 Leica laser scanning confocal microscope equipped with LAXS software and 63 × oil-immersion objectives.

### Mito Timer red plasmid and transfection

SiHa and CaSki CC cells underwent transfection with 2.5 µg of Mito Timer Red plasmid (Addgene, USA) with Lipofectamine™ 3000 (Thermo Fisher Scientific, USA) as transfection reagent. We used G418 (400 μg/mL for 2 weeks) for selecting population of cells expressing Mito Timer. Successful expression of the mitochondrial reporter was confirmed by fluorescence microscopy. We then exposed the established Mito Timer–expressing cells to BPA and analyzed mitochondrial morphology. Mitochondrial morphology was quantified from fluorescence images using the Mito-Morphology plugin in Fiji/ImageJ (Valente et al. 2017; Vojtova et al. 2024). The fluorescence images were transformed into 8-bit format, followed by thresholding to create binary images, and subjected to morphometric analysis. Parameters, including average branch length and circularity, were quantified to assess changes in mitochondrial network architecture and fragmentation. The average branch length was determined by measuring individual mitochondrial branches within cells and calculating the mean value, while circularity was used as an indicator of mitochondrial fragmentation (Adiga et al. 2023).

### Quantification of mitochondrial ROS (mtROS)

Mitochondrial superoxide levels were assessed in CC cells using Mito SOX Red staining following 48 h of exposure to two different concentrations of BPA in chambered cover glass systems. mtROS levels were estimated via the use of Mito SOX red dye (5 μM, 15 min; Ex/Em: ∼396/610 nm; Molecular Probes, USA). The plate was subsequently washed with phenol red-free DMEM and imaged via a confocal microscope. mtROS levels were quantified by measuring the average fluorescence intensity (*n* = 500 cells) using LASX software (Tomkova et al. 2018).

### Estimation of mitochondrial calcium (mtCa^2+^) levels

CC cells grown on 8-well chambered glass slides were treated with BPA at specified concentrations for 48 h. The cells were exposed to Rhod-2 AM (1 μM, 30 min, and *λ*ₑₓ/ₑₘ ~ 552/581 nm) containing 0.01% Pluronic F-127 and Hoechst-33342 (1 μg/mL, 5 min, and *λ*ₑₓ/ₑₘ ~ 350/461 nm) in HBSS (Ca^2^⁺/Mg^2^⁺-free). Cells were rinsed 3 × with HBSS (without Ca^2^⁺/Mg^2^⁺) prior to a 30-min de-esterification period (Adiga et al. 2023). Live cell imaging was performed via a confocal microscope, and the images were processed with LAS-X software. The mean fluorescence intensity from five hundred cells was quantified.

### Estimation of the mitochondrial mass (MM) and membrane potential (MMP)

The cells were grown on Ibidi polymer coverslips and treated with BPA at specified concentrations for 48 h. To measure the MM and MMP, we stained the cells (5 μM for 30 min) with nonyl acridine orange (NAO) (excitation/emission: ~ 469/525 nm; MedchemExpress, USA) and rhodamine-123 (excitation/emission: ~ 503/527 nm; MedchemExpress, USA), respectively. The cells were protected from light and maintained at 37 °C for half an hour. Cells washed with PBS and live imaged using a confocal microscope. The mean fluorescence intensity was calculated from five hundred cells with the help of LASX software (Adiga et al. 2023).

### Estimation of mitochondrial DNA depletion

mtDNA levels were evaluated following 48 h of treatment with BPA at two concentrations in chamber slide-cultured cells. We used 50 nM of Mito Tracker Red (ex/em: ~ 581/644 nm; Molecular Probes, USA) to label mitochondrial compartment and Pico Green (1:500 dilution; ex/em: ~ 480/520 nm; Molecular Probes, USA) to label DNA. Following a 30-min staining period at 37 °C in the dark, the cells underwent three PBS washes prior to analysis. We merged the Pico Green– and MitoTracker Red–stained images and performed colocalization analysis to identify mtDNA (specifically the Pico Green signal within MitoTracker Red-positive regions). The degree of mtDNA depletion was quantified by calculating the intensity of the green signal within Mito Tracker Red–stained regions (Adiga et al. 2023).

### Glucose uptake assay

The cultures were exposed to two doses of BPA continuously for 48 h. prior to analysis. The amount of glucose in the spent medium (10-µL samples obtained every 12 h for 48 h) was determined with a glucose assay kit (Agappe, India). The absorbance was recorded at 505 nm on a TECAN multimode microplate reader. To determine glucose consumption, the leftover glucose was deducted from the starting quantity and then related to the protein content (Adiga et al. 2023).

### Estimation of the oxygen consumption rate (OCR) and ATP

Cells at 50% confluence were treated with BPA in serum-free media for 48 h. Following 48 h of BPA exposure, adherent cells were harvested by trypsinization, counted, and normalized to equal cell numbers before OCR analysis using a Hansatech Oxygraph + instrument according to established protocols (Adiga et al. 2023).

After treatment, the cells were lysed using 1% Triton X-100. The ATP concentration was quantified with an ATP assay kit (Life Technologies, USA). For each measurement, we used 5 μL of cell lysate in a luminometer and normalized the results to the total protein content (Adiga et al. 2023).

### Estimation of antioxidant levels

Postexposure, adherent cells were extracted using 0.25% trypsin–EDTA, twice rinsed with ice-cold PBS (centrifugation at 2000 rpm, at 4 °C for 10 min), and lysed in RIPA-like buffer (10 mM Tris–HCl, pH 7.4, 30 mM KCl, 1% Triton X-100, and 1 mM EDTA) supplemented with protease inhibitors. The supernatants were used for enzyme assays.

GSH levels were quantified using the DTNB method by incubating lysates with DTNB (0.06 mM) in phosphate buffer (1 M, pH 8.0) for 30 min at 37 °C, followed by absorbance measurement at 412 nm. GSH levels were quantified using a standard curve and reported in relation to the total protein content (Rahman et al. 2006).

Catalase activity was quantified via a previously published protocol. In a microcentrifuge tube, we combined cell lysate (150 μL), H_₂_O_₂_ (600 μL), and PBS (750 μL). We added 150 μL of potassium dichromate allowed to react for 15-min at 37 °C. Catalase concentrations are determined from a standard curve generated from absorbance readings (240 nm) and expressed relative to total protein (Hadwan et al. 2024).

### Measurement of lipid peroxidation (LPR)

After 48 h of exposure to BPA, the cell pellets were mixed again with 200 μL of a TBA-TCA solution, which consists of 0.8% thiobarbituric acid in 20% trichloroacetic acid. This mixture was then heated at 95 °C for 30 min to form malondialdehyde-TBA adducts. The amount of malondialdehyde (MDA) formed was detected at 535 nm. The concentrations of MDA were assessed by creating a standard curve, which was then adjusted according to the total protein content of the cells. (Garcia et al. 2005).

### Localization of lipid droplets in mitochondria

Mitochondria-associated lipid droplets were analyzed via live cell confocal imaging. Cells were plated in Ibidi 8-well chamber slides and subsequently exposed to BPA in phenol red–free medium without FBS for 48 h. Following the PBS washes, the cells were sequentially kept in Rhodamine-123 (5 μg/mL, 30 min) for mitochondrial visualization, Nile Red (5 μg/mL, 30 min) for neutral lipid detection, and Hoechst 33342 (1 μg/mL, 5 min) for nuclear counterstaining. Using LAS X software, the colocalization of Nile red (*λ*_ex/em_ 552/636 nm) and rhodamine-123 (*λ*_ex/em_ 508/528 nm) signals was examined. The intensity of Nile red in the colocalized areas was used to quantify lipid droplet content (Adiga et al. 2023).

### Mitochondrial isolation

BPA-exposed and control cells (1 × 10⁷ cells) were trypsinized, washed with PBS (1000 × g, 10 min), and subjected to freeze‒thaw lysis in hypotonic solution (5 mM MgCl_₂_,10 mM Tris–HCl, and 10 mM NaCl, pH 7.6). The crude homogenate was reacted with sucrose (0.3 M final) and processed through dual centrifugation: initial clarification (600 × g, 15 min, 4 °C) followed by sedimentation of mitochondria (15,000 × g, 15 min, 4 °C). The pellet was frozen at −80 °C after being resuspended in 10 mM Tris–HCl with a pH of 7.4 (Liao et al. 2020).

### Western blot analysis of mitochondrial-specific markers

Mitochondrial pellets were treated with RIPA lysis buffer to extract mitochondrial proteins. Protein samples, each containing 45 μg, were separated using 10% SDS-PAGE and then moved onto nitrocellulose membranes (Bio-Rad, USA). Following a blocking step with 5% BSA, the membranes were dipped in primary antibodies against β-actin (1:3000; Cell Signaling Technology), PGC1α, TFAM, and DRP1 (1:3000; Cloud Clone Corp.) overnight at 4 °C. After a brief washing, membranes were immersed in HRP tagged secondary antibodies (1:5000; Cell Signaling Technology) for 1 h at RT. Protein bands were developed with Super Signal West Pico substrate (Thermo Fisher) and imaged in Image Quant LAS 4000 system (GE Healthcare) (Adiga et al. 2023).

### Cellular functional assays related to proliferation and migration

We conducted cell proliferation and migration assays to evaluate the biological impact of BPA on CC cells.

#### Cell proliferation assay

Around 20,000 cells were exposed to the desired concentrations of BPA in a 35-mm dish. Cell proliferation was assessed over a 5-day period by detaching cells on day 1 (24 h.), day 3 (72 h.), and day 5 (120 h.) with trypsin. Trypan blue staining–based cell counting using hemocytometer was used to count the viable cells (Khan et al. 2024).

#### Scratch assay

SiHa and CaSki cells were grown in a 6-well plate until they reached 80–90% confluency, after which they were serum-starved for 24 h. The line was drawn using a sterile micropipette tip (to produce a wound) at the center of each well, maintaining consistent length and width for each scratch. The cells were subsequently subjected to treatment with different BPA concentrations. Cells migrating into the scratched region were continuously monitored under a microscope (CKX41, Olympus, Japan). Cell migration into scratch areas were imaged at indicated time points. The rate of wound closure was analyzed using ImageJ/Fiji software (Khan et al. 2024).

### CC datasets and BPA interactive genes (BIGs)

To identify BPA-responsive mitochondrial genes in CC, we integrated data from three sources: (1) BPA-cancer associations from the Comparative Toxicogenomic Database (CTD) (Khan et al. 2024); (2) 1158 nuclear-encoded mitochondrial genes from MitoCarta2.0 (Meneur et al. 2021); and (3) genes that were differentially expressed (|log2FC|≥ 2) from TCGA-CESC via TACCO (http://tacco.life.nctu.edu.tw/). Venn analysis revealed consensus genes (BPA-responsive DENEMGs), which were subsequently analyzed for functional enrichment and pathway associations.

### Bioinformatic analysis

Using STRING V11.5 with a confidence score of 0.4, we developed a protein‒protein interaction network (PPIN) for the DENEMGs (Szklarczyk et al. 2019). Network construction and hub gene identification were performed in Cytoscape (v3.9.1) using the cytoHubba plugin. Based on maximal clique centrality (MCC) scores derived from the protein interaction network, the top 10 hub genes were identified and visualized (Chin et al. 2014). Molecular functions (MFs), cellular components (CCs), biological processes (BPs), and pathways associated with hub genes were identified using the Shiny GO v0.741 tool (Ge et al. 2020). The gene set cancer analysis (GSCA) tool was used to identify potential hub genes with drugs and immune cell abundance (Chen et al. 2022). Immune cell abundance correlations for the hub genes were computed through the GSCA database using TIMER algorithm-derived immune infiltration scores. Survival analysis was performed using the KM plotter, with optimal cutoff values determined by the tool’s algorithm, yielding log-rank *p*-values and hazard ratios (95% CIs) for both OS and DFS. Hub genes associated with cancer hallmarks were assessed via an online tool.

### Molecular docking analysis

The crystal structures of the target proteins were prepared by eliminating co-crystallized ligands and solvent molecules, followed by structural optimization with the Protein Preparation Wizard. Binding site grids were generated around specified active regions, and extra precision (XP) docking of BPA was performed via the Glide module. The binding free energy (ΔGbind) of ligand–protein complexes was determined through the MM-GBSA method ad part of the Prime module in the Schrödinger software suite. This calculation involved breaking down energy terms into electrostatic, van der Waals, and solvation components.

For MD using Desmond, an orthorhombic solvent box was prepared in the System Builder module using the OPLS3 force field parameters, the SPC water model, and a 0.15 M NaCl solution. Equilibration of the system was performed under the NPT ensemble (1 atm, 300 K), followed by an MD simulation for 100 ns. Trajectory analysis was performed using the Schrödinger software package for assessing root mean square deviation (RMSD) and root mean square fluctuation (RMSF).

### Statistical analysis

GraphPad Prism 9.0 (GraphPad Software, USA) was used to conduct statistical analyses. The data represent the mean ± SD of three independent experiments (*n* = 3 technical replicates per experiment). Group comparisons were analyzed using a two-tailed unpaired *t*-test or a one-way ANOVA, with statistical significance set at *p* < 0.05.

## Results

### BPA alters mitochondrial morphology in CC cells

**Fig. 1:**
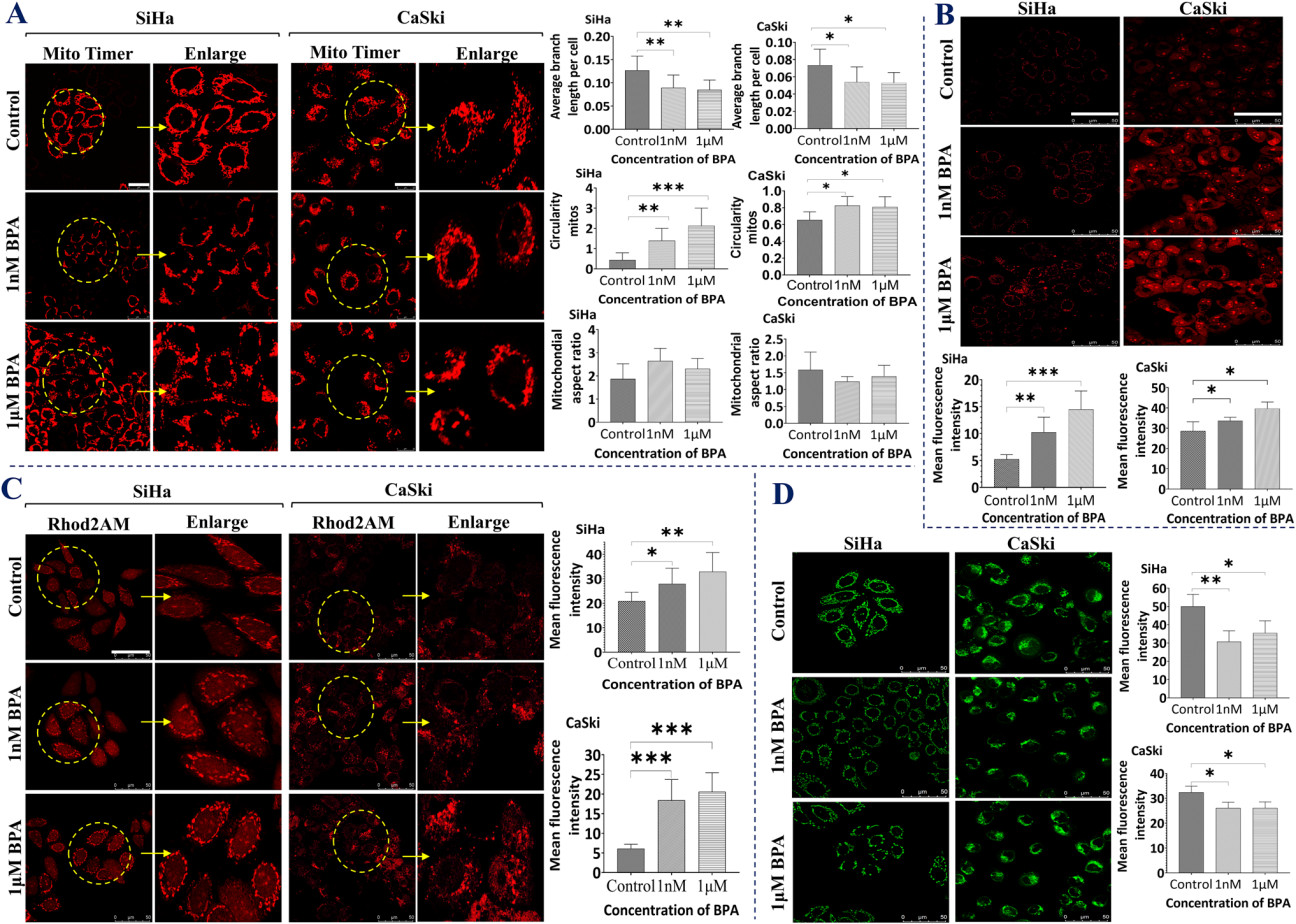
**A** BPA alters the structural appearance of mitochondria in cervical cancer (CC) cells. Representative confocal microscopy images of SiHa and CaSki cells transfected with the Mito Timer plasmid after BPA exposure (scale bar 25 μm). The corresponding bar graphs provide a quantitative assessment of mitochondrial dynamics and measurements of mitochondrial branch length, circularity, and aspect ratio. **B** BPA increases mitochondrial reactive oxygen species (mtROS) levels. Confocal images show mtROS accumulation in CC cells (scale bar 0–50 μm). The accompanying bar graphs indicate the mean fluorescence intensity derived from Mito SOX staining. **C** BPA increases mitochondrial calcium (mtCa^2^⁺) concentrations. Representative confocal microscopy images of SiHa and CaSki cells stained with Rhod2AM (scale bar 0–50 μm). The bar graphs show the mean fluorescence intensity for the mtCa^2^⁺ level. **D** BPA reduces the mitochondrial membrane potential (MMP) in CC cells. Confocal microscopy images show SiHa and CaSki cells stained with rhodamine 123 (scale bar 0–50 μm). The bar graphs depict the average MMP fluorescence intensity. Values are means ± standard errors with significance level denoted as ^*^p < 0.05, ^**^p < 0.01, and ^***^p < 0.001

Initial evaluation of the effects of BPA on mitochondria was conducted through live cell imaging of MitoTracker Red–stained SiHa and CaSki cells using confocal microscopy. Comparative analysis revealed distinct morphological alterations in mitochondrial networks between the BPA-exposed and control groups (Fig. 1A). We observed that in control cells, the mitochondria were elongated and filamentous, forming a well-developed filamentous network. Compared with no treatment, exposure to both 1 nM and 1 µM BPA significantly altered mitochondrial morphology, as evidenced by (1) decreased mitochondrial branch length, (2) reduced mitochondrial content, and (3) increased mitochondrial circularity (Fig. 1A; *p* < 0.05). Overall, BPA induced structural changes in mitochondria.

### BPA-induced mitochondrial ROS (mtROS) production

Previous studies have shown that abnormal mtROS levels contribute to CC cells (Kuo et al. 2022). mtROS analysis reveals significant BPA-induced oxidative stress. Following staining, the cells were imaged via confocal microscopy to quantify the fluorescence intensity as a measure of oxidative stress. Quantitative analysis revealed significant dose-dependent increases in fluorescence intensity following BPA exposure in both cell lines (Fig. 1B). In SiHa cells, the mean intensities increased from 5.213 (control) to 10.22 (1 nM BPA; *p* < 0.01) and 14.483 (1 μM BPA; *p* < 0.001). Similarly, the intensities of the CaSki cells increased from 28.629 (unexposed) to 33.65 (1 nM BPA; *p* < 0.05) and 39.672 (1 μM BPA; *p* < 0.01) (Fig. 1B). We showed that BPA treatment increased mitochondrial oxidative stress in CC cells.

### BPA increased mitochondrial calcium (mtCa^2+^) levels in CC cells

In CC, excessive mitochondrial Ca^2+^ uptake and subsequent imbalances promote carcinogenesis (Adiga et al. 2023; Chen et al. 2011). BPA effects on mtCa^2^⁺ were assessed via Rhod-2 AM staining in CC cells (Fig. 1C). Compared with no treatment, treatment with 1 nM or 1 μM BPA significantly elevated mitochondrial calcium levels in both CC cell lines (*p* < 0.05). In SiHa cells, the mean fluorescence intensity increased from 20.889 in the control group to 27.882 following 1 nM BPA exposure and further increased to 32.923 with 1 μM BPA treatment. Similarly, CaSki cells presented elevated mtCa^2+^ levels, with fluorescence intensities increasing from 6.114 in controls to 18.417 (1 nM BPA) and 20.560 (1 μM BPA) (Fig. 1C).

### BPA reduces mitochondrial membrane potential and mitochondrial mass in CC cells

**Fig. 2:**
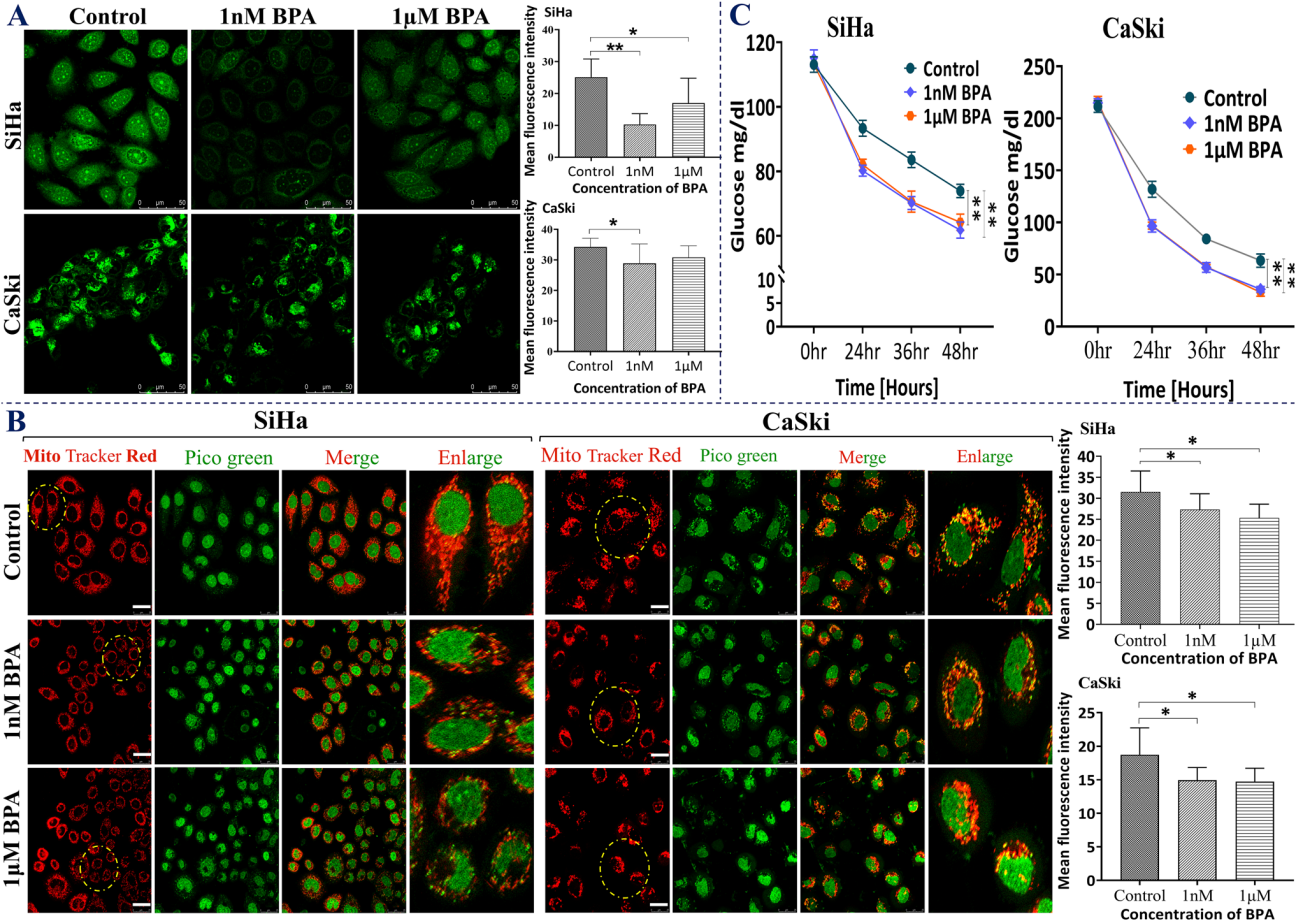
**A** BPA diminishes mitochondrial mass in CC cells. Representative confocal microscopy images of SiHa and CaSki cells stained with NAO (scale bar 0–50 μm). Bar graphs represent the mean fluorescence intensity of NAO. **B** BPA reduces mitochondrial DNA (mtDNA) depletion. Representative confocal microscopy images of SiHa and CaSki cells stained with Mito Tracker Red and co-stained with Pico Green. The bar graph shows the mtDNA fluorescence intensity. **C** BPA affects glucose consumption rates. A representative line graph shows glucose uptake rates in BPA-treated SiHa and CaSki cells. Values are means ± standard errors with significance level denoted as ^*^p < 0.05, ^**^p < 0.01, and ^***^p < 0.001

Changes in the MMP and MM can be observed in CC cells. To evaluate BPA-induced mitochondrial alterations, we assessed the MMP and MM via the fluorescent probes Rhodamine-123 (for the MMP) (Fig. 1D) and NAO (for the MM) (Fig. 2A), respectively. We detected significant decreases in MMP and MM in both cell lines after treatment with 1 nM or 1 μM BPA, relative to control cells. These data suggest that BPA exposure inhibits mitochondrial MMP and MM.

### BPA induced the mtDNA depletion in CC cells

Given previous reports of altered mtDNA depletion in various cancers, we next investigated whether BPA exposure changes mtDNA levels in our experimental system. To quantify BPA-induced changes in mtDNA depletion, cells were co-stained with PicoGreen (for nucleic acids) and MitoTracker Red (for mitochondria), followed by confocal microscopy imaging and colocalization analysis (Fig. 2B). Both nanomolar (1 nM) and micromolar (1 μM) BPA exposure induced significant (^*^p < 0.01) mtDNA depletion in CC cell lines.

### BPA increases the rate of glucose uptake in CC cells

Compared with normal cells, cancer cells consume more glucose (Bose et al. 2021). Environmental toxicants, especially cancer promoters, have been shown to promote glucose consumption. For this reason, we assessed the relationship between BPA exposure and glucose consumption. Compared with no treatment, BPA at concentrations of 1 nM or 1 μM significantly reduced extracellular glucose levels in SiHa and CaSki cell cultures after 48 h of exposure, indicating enhanced glucose utilization (*p* < 0.05) (Fig. 2C). This dose-dependent decrease indicates increased glucose utilization following BPA exposure.

### BPA exposure decreases antioxidant enzyme activity

**Fig. 3:**
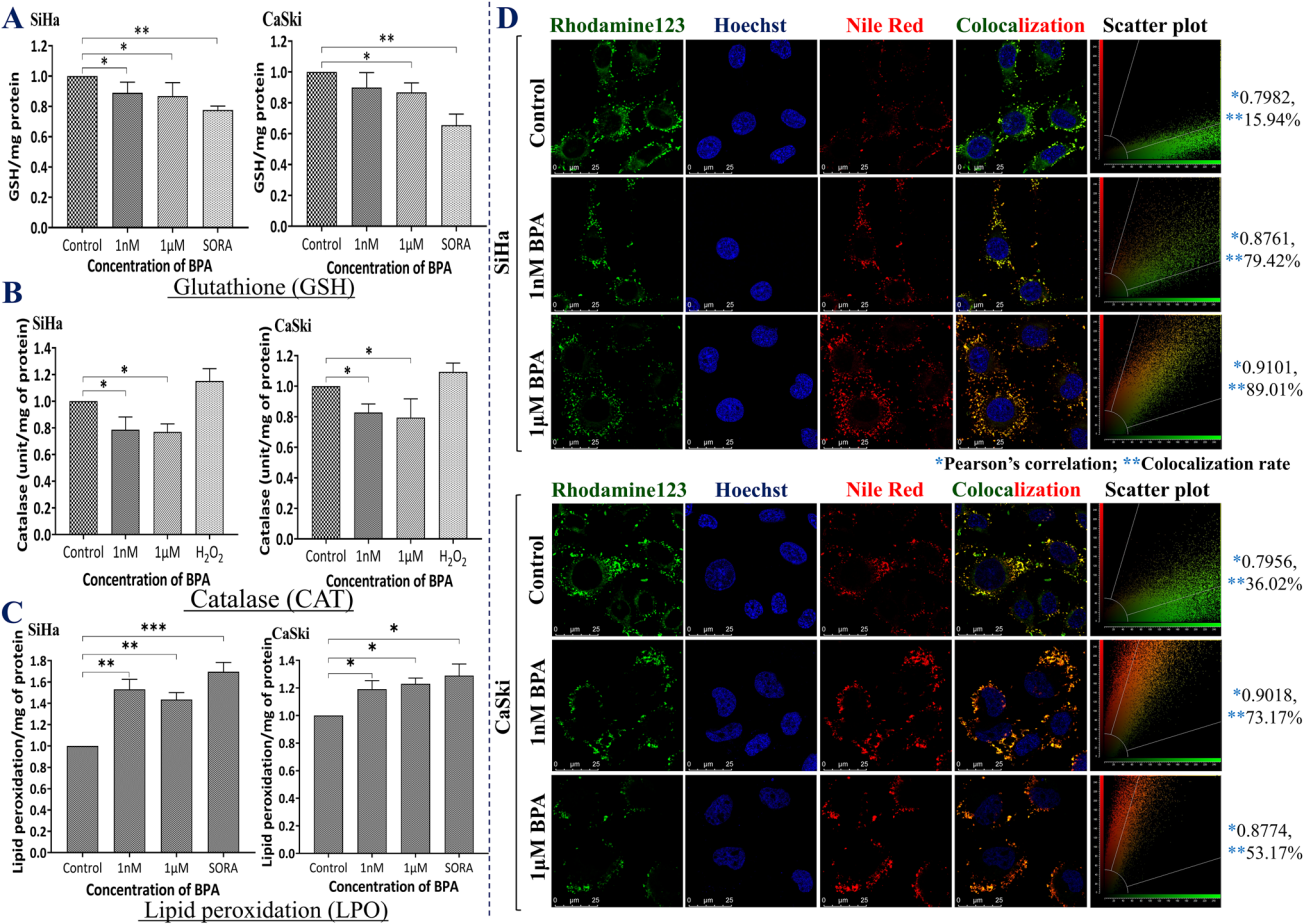
BPA reduces antioxidant activity and promotes lipid accumulation in the mitochondria of CC cells. **A** Glutathione (GSH), **B** catalase (CAT), and **C** malondialdehyde (MDA) levels in BPA-treated SiHa and CaSki cells. The bar graphs display absorbance units measured posttreatment. Values are means ± standard errors with significance level denoted as ^*^p < 0.05, ^**^p < 0.01, and ^***^p < 0.001. **D** Representative confocal microscopy images of SiHa and CaSki cells stained with rhodamine 123 and co-stained with Nile red and Hoechst. Images were captured using a 100 × oil immersion objective (scale bar 0–25 μM). Scatter plots provide the Pearson correlation coefficients

To assess the effect of BPA on antioxidant defense, we measured the activities of GSH and CAT in CC cell lines using spectrophotometric assays. BPA (1 nM) significantly depleted intracellular GSH levels in both cell lines. In SiHa cells, the GSH concentration was 0.89-fold (≈ 11% decrease) and 0.87-fold (≈ 13% decrease) that of the control at 1 nM and 1 µM, respectively. Similarly, in CaSki cells, the GSH concentration decreased 0.90-fold (≈ 10% decrease) and 0.87-fold (≈ 13% decrease), respectively, at the same concentration (Fig. 3A).

CAT activity was also markedly reduced upon BPA treatment. Compared with that of the control, the CAT activity of SiHa cells decreased 0.79-fold (≈ 21% decrease) and 0.77-fold (≈ 23% decrease) at 1 nM and 1 µM, respectively. In CaSki cells, CAT activity decreased by 0.83-fold (≈ 17% decrease) and 0.79-fold (≈ 21% decrease) at 1 nM and 1 µM BPA, respectively (Fig. 3B). These findings indicate that BPA impairs cellular antioxidant capacity by significantly decreasing GSH and catalase activities in a dose-dependent manner in CC cells.

### BPA induces lipid peroxidation in CC cells

LPR levels were measured in SiHa and CaSki cells to assess oxidative stress following treatment with 1 nM and 1 µM BPA and sorafenib (SORA). BPA treatment significantly elevated lipid peroxidation in SiHa cells (1.53-fold and 1.44-fold increases at 1 nM and 1 µM, respectively; Fig. 3C). In comparison with the control group, the SORA-treated group showed the greatest increase, at 1.70-fold, indicating substantial oxidative damage.

Similarly, in CaSki cells, BPA led to 1.19-fold and 1.23-fold increases in the LPR concentration at 1 nM and 1 µM, respectively (Fig. 3C). SORA-treated CaSki cells showed a further increase, reaching 1.29-fold higher than the control. These findings imply that BPA causes a dose-dependent increase in LPR, indicating increased oxidative stress in both CaSki and SiHa cells. In this investigation, sorafenib treatment was used as a positive control.

### BPA exposure increased lipid localization inside mitochondria

We evaluated the effects of BPA on lipid droplet‒mitochondrion interactions, as lipid droplet accumulation is closely linked to carcinogenesis. We performed confocal microscopy to quantitatively assess lipid droplet localization within the mitochondria of CC cells by co-staining with Rhodamine-123 and Nile Red (lipid droplet probes) (Fig. 3D). There were substantial increases in lipid droplet localization within the mitochondria of both CC cells under the influence of BPA. The quantification of colocalization showed a statistically significant enhance in mitochondrial lipid droplets in SiHa cells after BPA exposure, in a concentration-dependent manner. The control cells exhibited a baseline association (*r* = 0.798, 15.94% colocalization), which then increased upon exposure to 1 nM BPA (*r* = 0.876, 79.42% colocalization; *p* < 0.01) and 1 μM BPA (*r* = 0.910, 89.01% colocalization; *p* < 0.001). A similar dose-dependent pattern was observed in CaSki cells. Unexposed CaSki cells showed a baseline association (Pearson’s *r* = 0.796, 36.02% colocalization), which increased sharply with 1 nM BPA exposure (*r* = 0.902, 73.17%; *p* < 0.001). Interestingly, 1 μM BPA treatment resulted in a partial reversal (*r* = 0.877, 53.17%; *p* < 0.05 vs. 1 nM). Thus, BPA increases lipid droplet accumulation in CC cells (Fig. 3D).

### BPA exposure decreases the OCR in CC cells

**Fig. 4:**
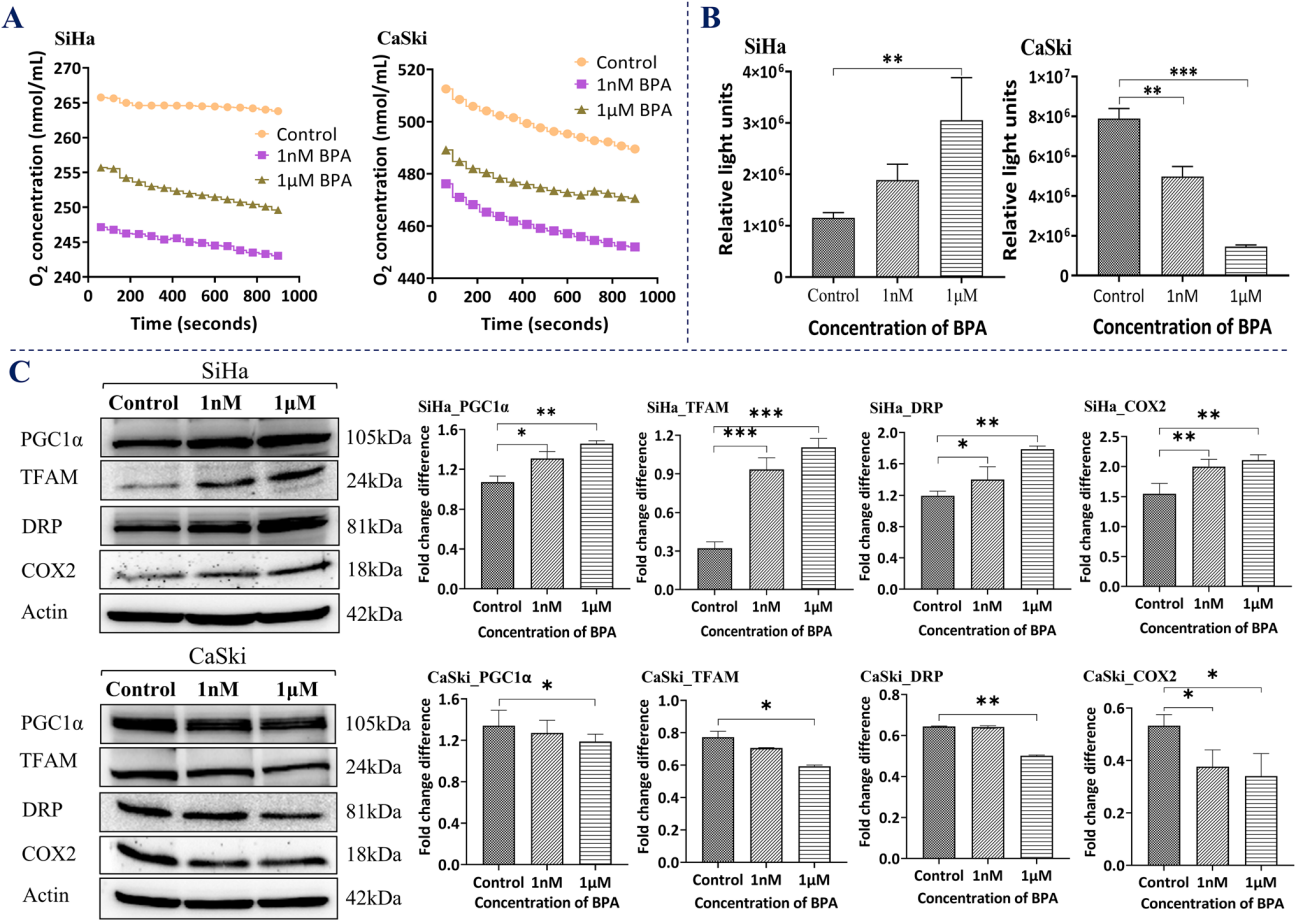
**A** Line graph representing the oxygen consumption rate (OCR) in SiHa and CaSki cells exposed to BPA. **B** BPA-treated CC cells exhibit changes in ATP production. The bar graphs present ATP levels in CC cells. **C** BPA affects mitochondrial biogenesis and dynamics gene expression. Western blot images showing mitochondrial gene expression in BPA-exposed SiHa and CaSki cells. Densitometric analysis illustrates the relative fold change in gene expression, normalized to that of actin, between the treated and control groups. Values are means ± standard errors with significance level denoted as ^*^p < 0.05, ^**^p < 0.01, and ^***^p < 0.001

Given the critical role of mitochondrial dysfunction in cancer metabolism, we evaluated the impact of BPA on oxygen consumption rates (OCRs) in both CC cells following exposure to 1 nM and 1 µM BPA (Fig. 4A).

Control SiHa cells were characterized by steady OCR levels across all tested time points, beginning at 265.76 pmol/min at 60 s and decreasing to 263.79 pmol/min at 900 s. On the contrary, cells exposed to 1 nM BPA had significantly decreased OCR values (from 247.14 to 243.04 pmol/min), while OCR values for 900 s decreased even more (from 255.70 to 249.62 pmol/min) (Fig. 4A).

In agreement with the results for SiHa cells, there were similar effects of BPA on mitochondria in CaSki cells. The control cells showed a high OCR baseline with a value ranging from 512.56 to 489.51 pmol/min after 900 s. Exposure to 1 nM BPA caused a drop in OCR value from 476.19 to 451.97 pmol/min, while exposure to 1 µM BPA reduced the OCR from 489.16 to 470.65 pmol/min within 900 s (Fig. 4A).

### BPA differentially regulates ATP production in SiHa and CaSki cells

To evaluate the effects of BPA on cellular bioenergetics, intracellular ATP levels were quantified in CC cells following exposure to 1 nM and 1 µM BPA for 48 h. BPA treatment at both 1 nM and 1 μM induced opposing effects on ATP synthesis, stimulating ATP production in SiHa cells and inhibiting it in CaSki cells compared with control cells (Fig. 4B). ATP levels increased in SiHa cells but decreased in CaSki cells following BPA exposure.

### BPA alters mitochondrial biogenesis and dynamics-associated protein expression

Western blot analysis was performed to test mitochondrial protein expression in BPA-exposed SiHa and CaSki cells (Fig. 4C). Genes such as PGC1α, TFAM, and DRP1 were analyzed. BPA exposure induced dose-dependent upregulation of mitochondrial biogenesis markers (PGC1α and TFAM) and fission-related proteins (DRP1 and COX2) in SiHa cells, with significant increases observed at both 1 nM and 1 μM BPA (*p* < 0.05). In contrast to SiHa cells, CaSki cells exposed to 1 nM and 1 µM BPA exhibited significant downregulation of the expression of mitochondrial regulatory proteins, including biogenesis markers (PGC1α and TFAM) and fission-related factors (COX2 and DRP1), compared with those in untreated controls (Fig. 4C).

### BPA increases the proliferation of CC cells

**Fig. 5:**
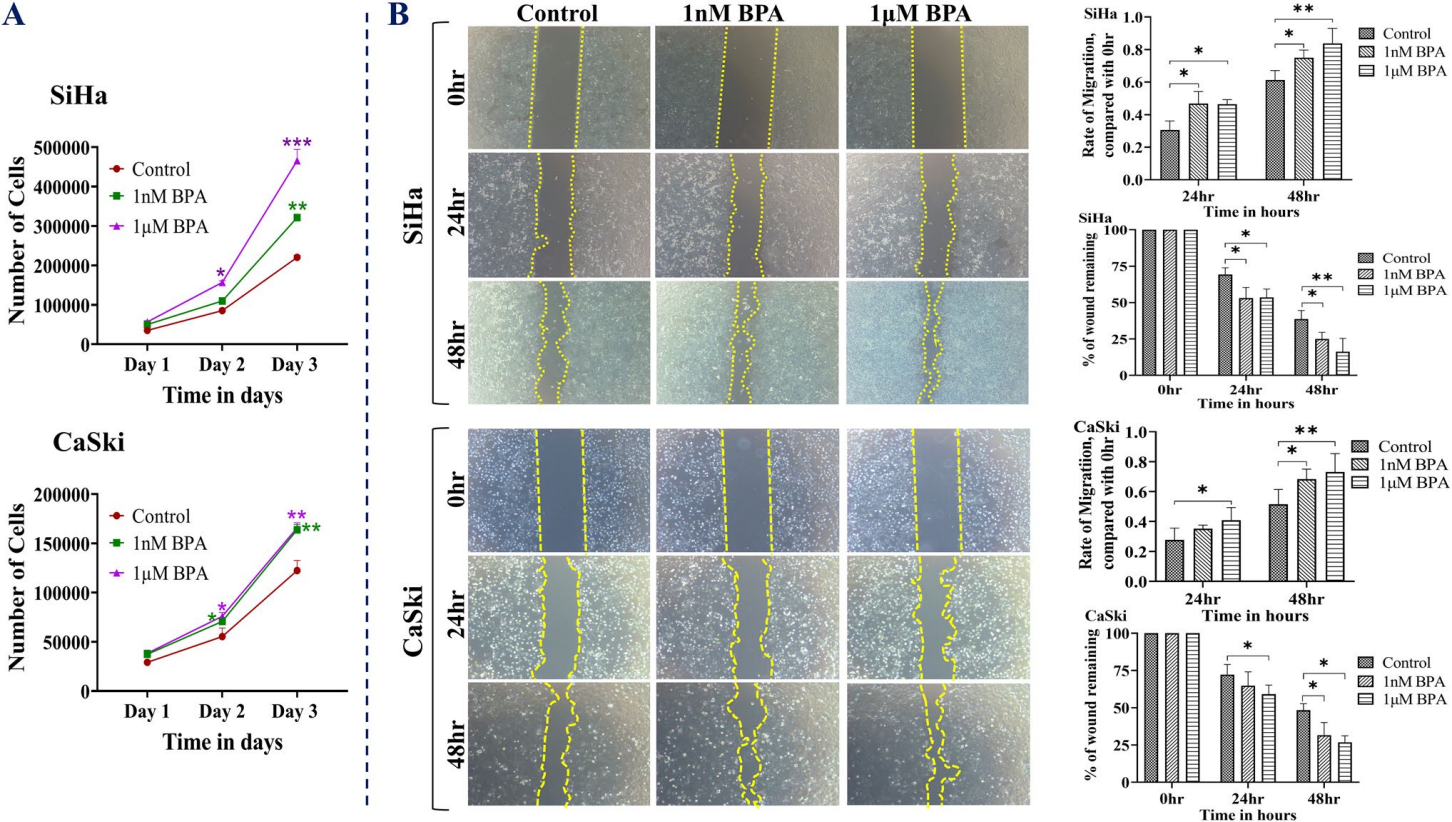
BPA promotes proliferation and migration of CC cells. **A** Assay of cell proliferation illustrating the impact of BPA at concentrations of 1 nM and 1 μM on the proliferation of SiHa and CaSki cells across a period of five days. Cell proliferation was determined by viable cell counting using the trypan blue exclusion method. **B** Representative images of the scratch assay (left) and quantification of migration rate (% wound closure) and percentage of wound remaining (right) following BPA exposure. Differences relative to the control group were considered statistically significant at ^*^p < 0.05 and ^**^p < 0.01, as determined by one-way ANOVA followed by Tukey’s post hoc test

To evaluate the impact of BPA on cell proliferation, CC cells were exposed to 1 nM and 1 μM BPA, and viable cell counts were determined over five days. BPA treatment significantly accelerated the proliferative capacity of both cell lines compared with the untreated controls (Fig. 5A). The increased cell number was observed in a concentration- and time-dependent effects, with the highest proliferative effect recorded at 1 μM BPA on day 5 in both SiHa and CaSki cells.

### BPA exposure increases CC cell migration

To investigate the influence of BPA on CC cell migration, a scratch assay was. BPA exposure significantly accelerated the migration capacity of both cells compared with untreated controls (Fig. 5B). At 48 h, the percentage of wound closure in SiHa cells enhanced from 61.29% in the control group to 75.02% and 83.83% following exposure to 1 nM and 1 μM BPA, respectively. Correspondingly, the remaining wound area decreased from 38.71% in the control group to 24.98% and 16.17% in the 1 nM and 1 μM BPA-treated groups, respectively. Similarly, BPA treatment increased the migratory capacity of CaSki cells. At 48 h, wound closure enhanced from 51.62% in control cells to 68.38% and 73.13% following treatment with 1 nM and 1 μM BPA, respectively, while the remaining wound area decreased from 48.38 to 31.62 and 26.87%, respectively. These results demonstrate that BPA significantly promotes the migratory ability of CC cells in a concentration-dependent manner (Fig. 5B).

### Identification of BPA-interacting genes (BIGs) in CC via in silico analysis

BPA-associated CC genes were identified through CTD, MitoCarta, and TCGA integration. Initially, a list of 1159 nuclear-encoded mitochondrial genes was retrieved from the MitoCarta 2.0 database. In parallel, 2031 DEGs associated with CC were obtained from the TCGA-CESC (The Cancer Genome Atlas–Cervical Squamous Cell Carcinoma and Endocervical Adenocarcinoma) dataset. The two gene sets were compared via Venny 2.1, a tool for identifying overlapping genes through Venn diagram–based analysis. This comparison revealed 52 nuclear-encoded mitochondrial genes that were differentially expressed in both datasets. A comprehensive analysis of the CTD revealed 3760 unique gene interactions associated with both bisphenol A exposure and cancer pathogenesis. The intersection between the 52 mitochondrial DEGs and the CTD-derived gene list was then analyzed. This integrative approach identified 18 candidate genes that were both differentially expressed in CC and potentially responsive to BPA exposure. These genes include *ABCA9, ABCD2, ACACB, ACSM1, BCL2, CDC25C, CLIC4, CPT1B, EFHD1, FASN, HK2, IDH2, MAOB, NCEH1, PDK4, PMAIP1, SLC25A10,* and *TUBB3*. These 18 genes represent a set of putative BPA-interacting genes that may be involved in mitochondrial dysfunction and pathways relevant to CC pathogenesis, warranting further experimental validation.

### PPIN analysis and hub gene identification

**Fig. 6:**
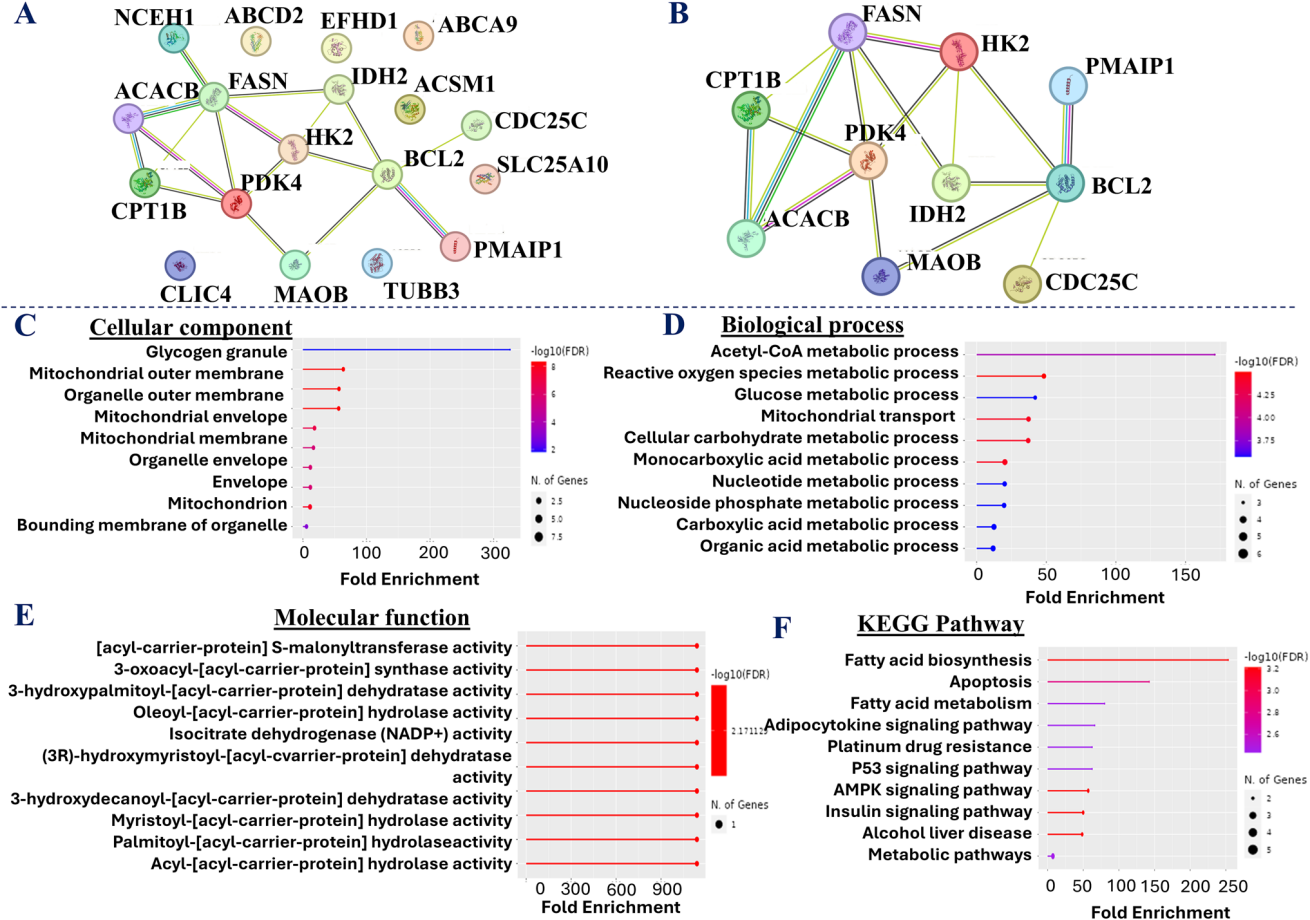
Functional enrichment and pathway analysis of BPA-responsive genes. **A** Protein‒protein interaction network (PPIN) for 18 interacting genes. **B** 10 hub genes identified within the PPIN. **C** Top 10 enriched cellular components linked to BPA-responsive genes. **D** Top 10 enriched biological processes associated with BPA-responsive genes. **E** Top 10 enriched molecular functions related to BPA-responsive genes. **F** Top 10 enriched pathways influenced by BPA-responsive genes

PPIN of the BIGs, generated via the STRING database, revealed key regulatory genes within the network. The PPIN comprised 18 nodes and 17 edges, indicating strong functional associations, with a significant enrichment *p*-value of 1.57 × 10⁻⁸ (Fig. 6A). Topological analysis using the cytoHubba plugin identified 10 high-confidence hub genes in the BPA-interacting network: *FASN, CPT1B, HK2, PMAIP1, BCL2, CDC25C, MAOB, ACACB, PDK4,* and *IDH2.* The constructed hub gene network comprised 10 nodes and 16 edges, with an enrichment *p*-value of 1.03 × 10⁻⁹ (Fig. 6B). Overall, these results emphasize key genes whose expression is potentially linked with BPA.

### Functional enrichment analysis (FEA)

**Fig. 7:**
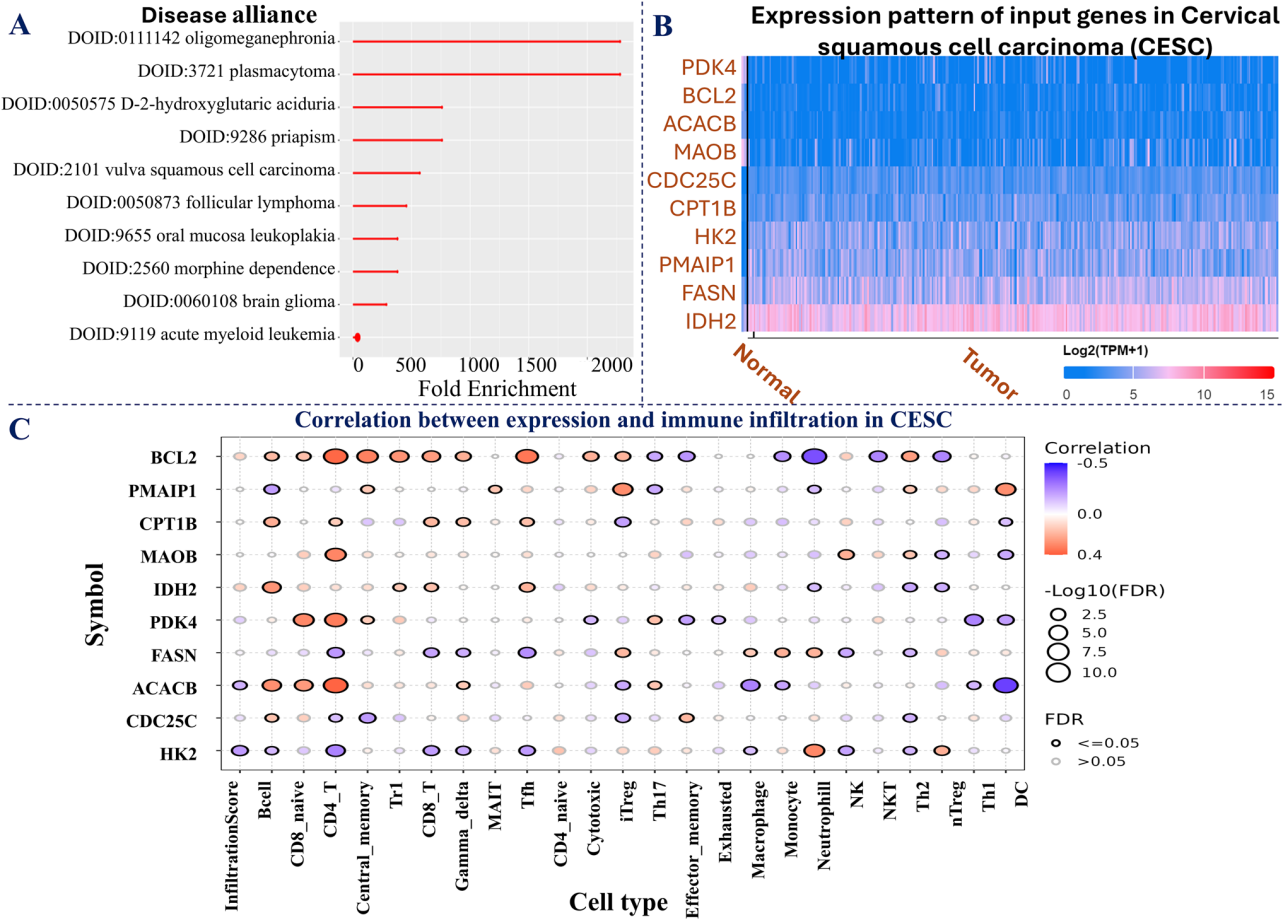
**A** The top 10 disease associations enriched among BPA-responsive genes. **B** Expression profiles of hub genes in cervical squamous cell carcinoma (CESC). **C** Bubble plot summarizing the correlations between the mRNA expression of the input genes and that of 25 immune cell types associated with tumor infiltration in selected cancers

FEA of the BIGs (Fig. 6C–F) revealed significant associations across multiple biological categories. The top 10 enriched CCs (Fig. 6C), biological processes (Fig. 6D), molecular functions (Fig. 6E), KEGG pathways (Fig. 6F), and disease alliances (Fig. 7A) were identified. Notably, several enriched cellular components, including the mitochondrial outer membrane, the mitochondrial envelope, and glycogen granules, underscore a strong mitochondrial association. Given the pivotal role of mitochondria in metabolic reprogramming, these findings demonstrate that BPA-responsive genes dysregulate key oncogenic pathways, potentially driving cervical carcinogenesis through mitochondrial dysfunction.

### Overall survival analysis of hub genes

Prognostic evaluation of the ten hub genes was carried out using Cox proportional hazards regression and Kaplan‒Meier survival analysis of the TCGA-CESC data (Supplementary Fig. 1). Among these genes, FASN (HR = 2.99, *p* = 1.4 × 10⁻^5^) and HK2 (HR = 2.18, *p* = 0.0018) were significantly associated with reduced overall survival, suggesting that these genes may have potential negative prognostic effects in CC. Conversely, elevated expression of *BCL2* (HR = 0.40; *p* = 0.00017), *IDH2* (HR = 0.39; *p* = 0.00011), and *CDC25C* (HR = 0.53; *p* = 0.0066) correlated with improved survival. The expression of *PDK4, ACACB, CPT1B, MAOB,* and *PMAIP1* was not significantly associated with overall survival. These findings suggest that selected BPA-responsive hub genes may have potential prognostic relevance in CC.

### Drug‒gene interactions and immune infiltration

**Fig. 8:**
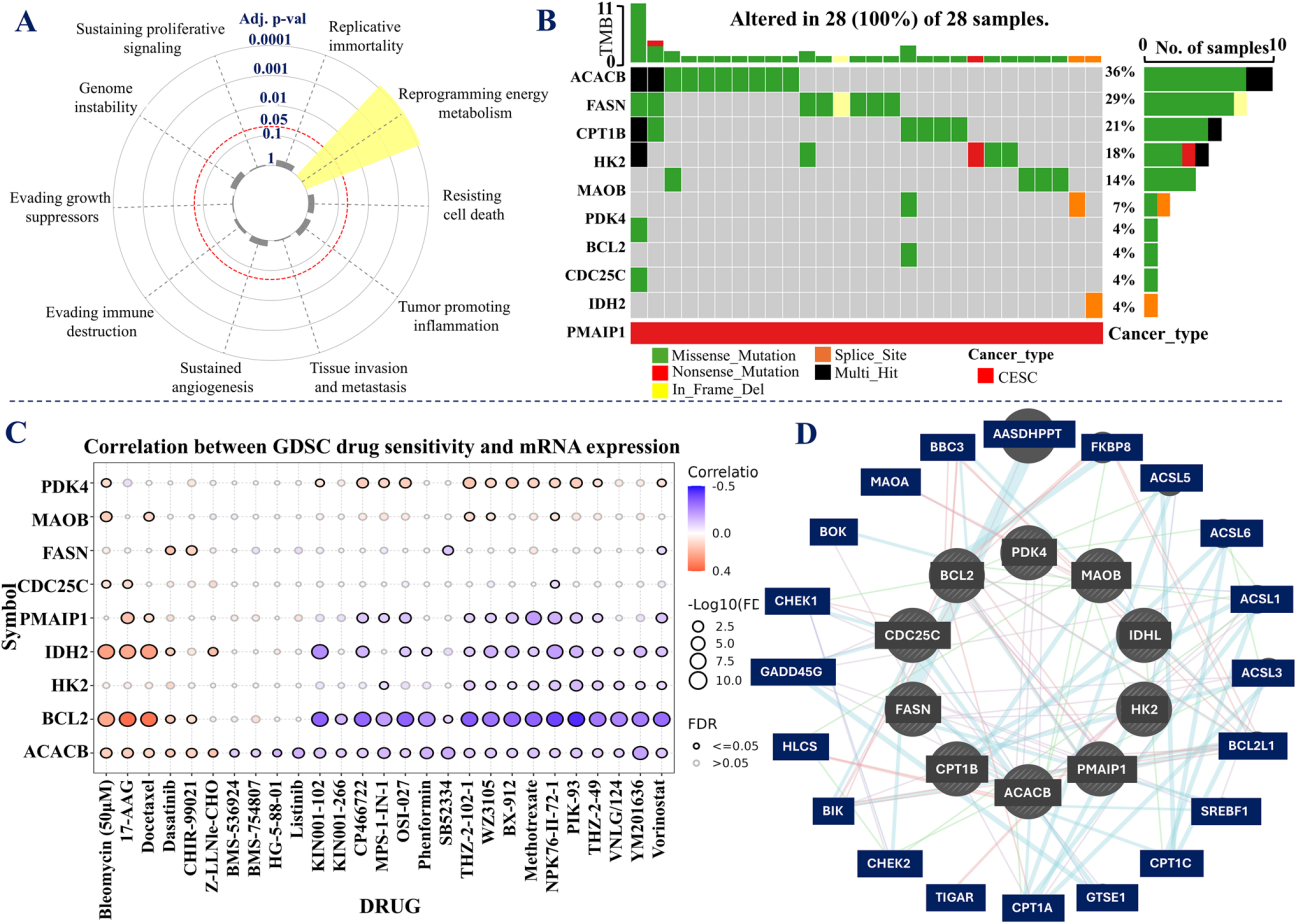
**A** Functional characterization of BPA-interacting genes (BIGs) in CC and their connections to cancer hallmark pathways. **B** Genetic alteration analysis revealed that 100% of the examined CC samples harbor alterations in at least one BIG. **C** Bubble plot illustrates correlations between hub genes and drug sensitivity, as determined by the Genomics of Drug Sensitivity in Cancer database. The relationship was analyzed using Pearson correlation

To explore the potential association between BIG expression and immune cell infiltration in CC, the TCGA-CESC and GSCA cohorts were used. Our findings revealed that BPA treatment significantly influenced immune cell and infiltration gene expression (Fig. 7C). We further analyzed drug–gene interactions for the top 10 BIGs. This analysis revealed 9 genes targeted by 27 drugs (Fig. 8C), indicating that multiple opportunities for therapeutic intervention in the tumor microenvironment, together with immune cell infiltration, plays a critical role in regulating tumor progression, patient prognosis, and responses to therapeutic interventions (Wang et al. 2023).

### Hallmark pathways, genetic alterations, and interaction networks

We next assessed potential associations among BIGs, cancer hallmark pathways, and genetic alterations to better understand their roles in CC. Hallmark enrichment analysis revealed significant involvement of BIGs in the reprogramming of energy metabolism (Fig. 8A; *p*-value < 0.0001). Mutations profiling showed that all samples of CC had mutations in at least one BIG (Fig. 8B). Interaction network mapping identified extensive crosstalk among the hub genes and their co-expressed partners (Fig. 8D).

### Molecular docking and binding free energy

**Fig. 9:**
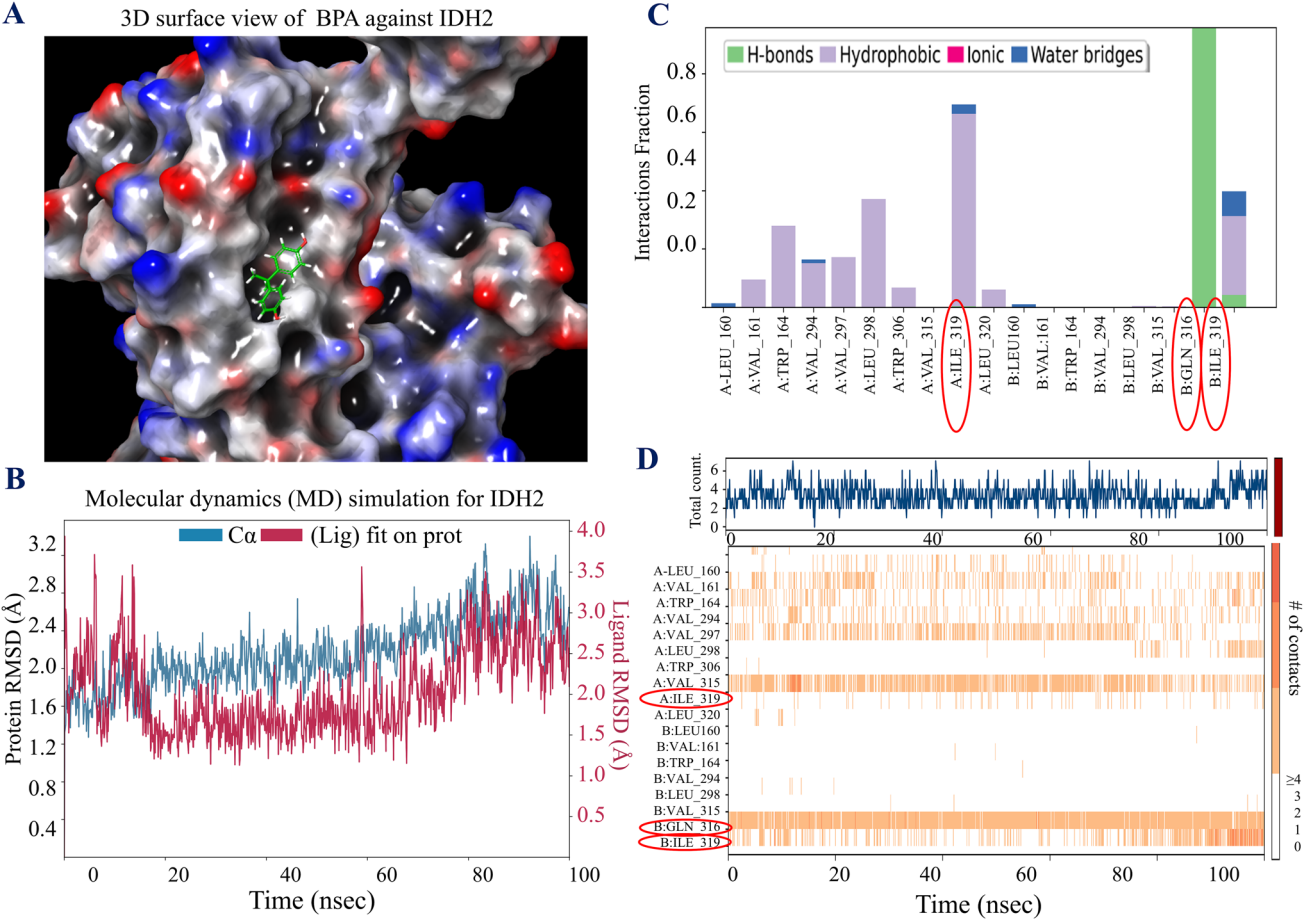
In silico analysis of BPA and IDH2 protein interaction. **A** Three-dimensional surface representation of the binding interaction. **B** Root-mean-square deviation (RMSD) plots for assessing structural stability. **C** Histogram of protein‒ligand interaction frequencies. **D** Timeline analysis of protein–ligand interactions

The three-dimensional structures of the ten hub proteins were obtained from the RCSB (PDB) (PDB ID: *FASN*−3HHD, *IDH2*−5I96, *BCL2*−5VAU, *PDK4*−7EBB, *ACACB*−3JRX, and *CDC25C*−3OP3) and the AlphaFold database (AF IDs: *HK2*-Q92523, *CPT1B*-P52789, and *PMAIP1*-Q13794). Binding sites were identified using SiteMap analysis, and the top-scoring sites were selected for the docking studies. Molecular docking of BPA was performed in Glide’s extra precision (XP) mode, revealing strong binding affinities for *IDH2* (docking score −7.384 kcal/mol), *MAOB* (−6.29 kcal/mol), and ACACB (−5.972 kcal/mol). Notably, no viable poses were generated for *PMAIP1* in XP mode. Detailed interaction profiles, including docking scores, glide energies, MM-GBSA ΔG binding values, and nonbonding interactions, are presented in Table 1, and a 3D surface view of BPA with the *IDH2* protein is shown in Fig. 9A.

**Table 1:**
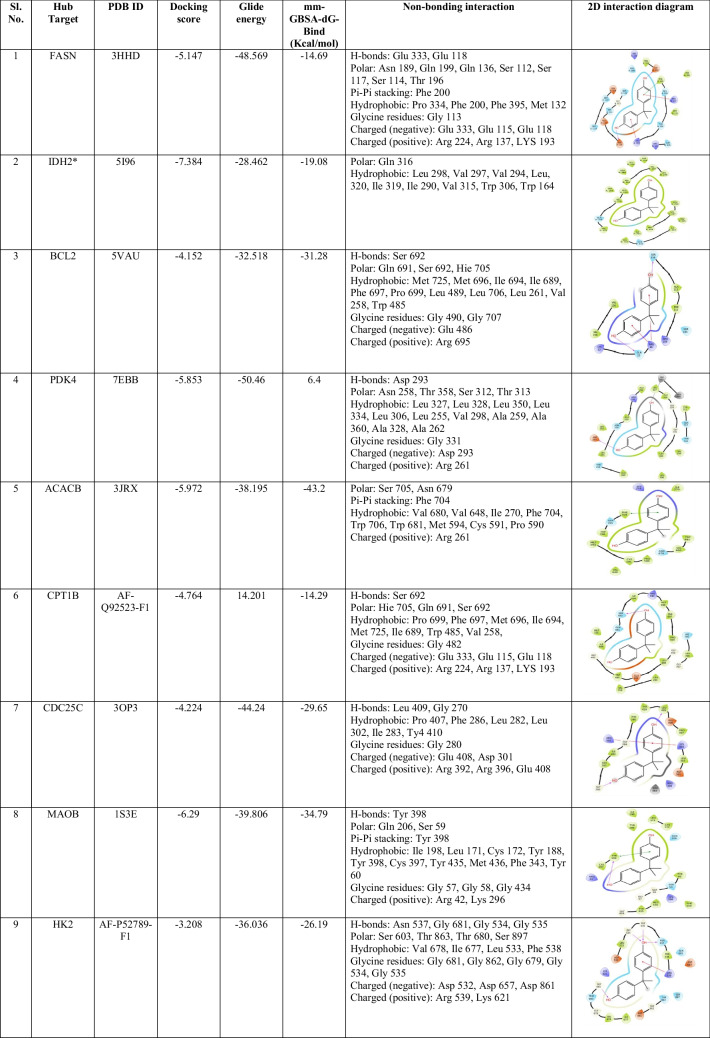
Results of glide score, molecular mechanics with generalized birth and surface area solvation (MM-GBSA) assay for top 10 hub genes against BPA

Bisphenol A: (Pubchem ID: 6623)

BPA Isomeric SMILES: CC(C)(C1 = CC = C(C = C1)O)C2 = CC = C(C = C2)O

Software: Schrodinger

Binding free energies were calculated via the MM-GBSA module. We observed strong interactions between BPA and all hub targets except *PDK4*. MM-GBSA calculations revealed *HK2* as the strongest BPA-binding target (Δ*G* = −34.79 kcal/mol), followed by *IDH2* (−28.46 kcal/mol) and *FASN* (−25.91 kcal/mol). Notably, PDK4 exhibited significantly weaker binding (−14.79 kcal/mol), whereas the other targets showed binding energies of less than −20 kcal/mol, suggesting stable complex formation.

### Molecular dynamics (MD) simulation analysis

Molecular dynamics simulations (100 ns) confirmed the stable binding of BPA to IDH2. Postsimulation analyses included a comprehensive evaluation of ligand-binding site dynamics, including the RMSD and RMSF. The system reached equilibrium with stable conformational dynamics, as evidenced by average RMSD values of 2.5 Å (protein backbone) and 3.2 Å (ligand). In comparison, RMSF analysis indicated moderate residue flexibility (2.9 Å) throughout the 100-ns simulation (Fig. 9B). BPA maintained persistent interactions (> 90% simulation time) with the *IDH2* binding pocket, forming stable hydrophobic interactions with residues A: ILE_319, A: LEU_298, and B: ILE_319 and polar interactions with B: GLN_316 and B: ILE_319 (Fig. 9C). Protein–ligand contact interactions and secondary structure element (SSE) dynamics were also assessed (Fig. 9D).

## Discussion

The present study provides experimental proof that BPA can induce profound structural and functional alterations in the mitochondria of CC cells. We previously reported increased proliferation, invasion, and migration of CC cells following BPA treatment (Khan et al. 2024). Present study demonstrates that BPA exposure (i) enhances mitochondrial-associated lipid droplet accumulation indicating its potential role in metabolic reprogramming; (ii) changes in mitochondrial morphology, oxidative stress, Ca^2+^ overload, and bioenergetic dysfunction; (iii) cell type specific mitochondrial responses suggesting the importance of tumor heterogeneity in environmental carcinogenesis; (iv) identification of BPA-responsive mitochondrial hub genes with prognostic significance in CC; and (v) potential direct binding of BPA with IDH2, suggesting its interaction with metabolic enzymes through molecular docking. Thus, these findings support the existence of a BPA–mitochondrial axis in CC.

Altered mitochondrial morphology has been observed during progression of diverse human cancers (Ma et al. 2020). Our study revealed that compared with control cells, BPA-exposed CC cells presented fragmented mitochondria, increased circularity, and reduced branch length and content. A decrease in the MMP and MM upon BPA exposure indicates mitochondrial depolarization and compromised mitochondrial integrity. These changes are consistent across both cell lines, suggesting strong induction of mitochondrial dysfunction in CC cells. We observed mtDNA depletion in response to BPA exposure. This observation is consistent with our earlier study that showed mtDNA depletion in CC samples compared with normal samples (Kabekkodu et al. 2014). A reduction in mtDNA may contribute to altered oxidative phosphorylation and genomic instability (Hebert et al. 2010, Lee and Wei 2009). Overall, we showed that BPA can disrupt mitochondrial structure and reduce the mtDNA depletion in CC cell lines.

Oxidative stress and Ca^2+^ overload are interconnected in several cancers. Oxidative stress results from a disrupted equilibrium between ROS production and cellular antioxidant defense mechanisms (Jomova et al. 2023, Remigante and Morabito 2024). Oxidative stress has been shown to induce Ca^2+^ overload, which may cause mitochondrial stress (McElnea et al. 2011, Peng and Jou 2010). Although Ca^2^⁺ signaling is known to regulate apoptosis, few reports have established its tumor-promoting capacity (Li et al. 2024; Wu et al. 2020). However, few studies have shown that excessive Ca^2+^ may induce apoptosis (Gielecinska et al. 2024; Pinton et al. 2008). We identified BPA as a promoter of oxidative stress and Ca^2+^ overload (Khan et al. 2024). Previous research revealed that BPA induces CC cell migration via Ca^2^⁺-mediated nuclear translocation of β-catenin (Khan et al. 2024). Notably, the tested BPA concentrations did not trigger apoptotic cell death in either the SiHa or CaSki cell lines (data not shown). Instead, it promoted cell proliferation in both cell lines (Khan et al. 2024). The increase in mtCa^2^⁺ levels indicates that BPA could activate the mitochondrial permeability transition pore. Cancer cells often exploit this pathway to avoid apoptosis, thereby enabling continuous proliferation. Findings from previous studies, including ours, suggest that BPA-induced mitochondrial dysfunction may contribute to the more aggressive phenotypes observed in CC cells, such as increased proliferation and migration.

Redox imbalances play important roles in carcinogenesis (Xian et al. 2019). Our results demonstrate that BPA disrupts redox homeostasis in CC cells, as evidenced by depleted GSH/CAT antioxidant defenses, which are accompanied by elevated ROS levels and lipid peroxidation. BPA treatment may increase intracellular mtROS levels by suppressing GSH and CAT activity. Given that ROS directly triggers lipid peroxidation, the elevated lipid peroxidation after BPA exposure could stem from both reduced GSH/CAT activity and heightened ROS production.

The accumulation of lipid droplets following BPA treatment, a key finding of this study, suggests possible metabolic rewiring. Lipid droplet accumulation is now considered a significant factor driving cancer (Li et al. 2020). Lipid droplets act as substrates that provide both lipids and energy for highly proliferative tumor cells (Jin et al. 2023; Li et al. 2020). This study reports for the first time accumulation of lipid droplets in mitochondria as a result of BPA exposure in SiHa and CaSki cells. Lipid droplets are known to protect cancer cells from oxidative stress–induced damage. Nevertheless, the role of increased lipid droplet accumulation upon BPA exposure warrants further investigation.

Changes in intracellular ATP levels, lipid droplet accumulation, and glucose uptake upon BPA exposure suggest a possible role for BPA in metabolic reprogramming. In SiHa cells, BPA treatment increased intracellular ATP levels and mitochondrial biogenesis factors despite mitochondrial damage, suggesting that BPA attempts to maintain oxidative phosphorylation under BPA stress. However, CaSki cells presented dose-dependent reductions in intracellular ATP levels and mitochondrial biogenesis factor levels, suggesting a possible shift toward glycolytic metabolism. The ATP response observed in this particular cell type aligns with findings from an earlier study involving triple-negative breast cancer cells (Pelicano et al. 2014): BPA exposure increased intracellular ATP levels in MDA-MB-231 cells but decreased them in BT-549 cells. These different responses by each cell line toward the same amount of BPA emphasize the importance of taking into consideration the heterogeneity of tumors when analyzing responses to environmental carcinogens. The presence of BPA upregulated the glucose uptake in both the cell lines, indicating an increased glycolysis flux, which was one of the strategies used by cancer cells to grow and survive (Al Hasawi et al. 2014; Kooshan et al. 2024). BPA exposure simultaneously increased both glucose uptake and lipid droplet accumulation in all tested cell lines. These changes suggest that BPA may play a role in metabolic reprogramming. Western blot and qRT‒PCR analyses demonstrated that BPA impaired genes related to mitochondrial biogenesis (*PGC1α, TFAM, DRP1*, and* SIRT3*), with opposite effects in SiHa and CaSki cells. These findings propose that BPA may exert context-dependent effects on energy metabolism and mitochondrial turnover.

Strong binding affinities between BPA and metabolic enzymes (IDH2, HK2, and FASN) were identified by molecular docking and dynamic simulation, suggesting a potential influence on metabolism. One important enzyme in the TCA cycle is IDH2. The observed metabolic changes may be explained by BPA’s persistent interaction with IDH2. Furthermore, our results showing elevated lipid droplet accumulation are consistent with the prediction that BPA binds to FASN (lipogenesis) and ACACB (fatty acid oxidation).

While our findings provide evidence that BPA disrupts mitochondrial function in CC cells, our study has several limitations. The present study addresses the use of in vitro models, fixed doses and times, and mechanistic gaps. While we noted increased glucose consumption in BPA-exposed CC cells, we did not investigate the expression of glucose transporters (GLUT family). Future studies examining GLUT expression and its membrane translocation would provide mechanistic insights into BPA-enhanced glucose uptake in CC cells. The hub genes presented in the study are derived from in silico analysis and require validation. Lack of functional validation using in vivo models is a limitation of our study. Even so, we demonstrated that BPA can affect mitochondria in CC cells. Research on these gaps in future work may clarify the broader implications of BPA in CC.

## Conclusion

In conclusion, our research shows that BPA-exposed CC cells exhibit structural, functional, and metabolic impairments, accompanied by disrupted mitochondrial homeostasis. Further research using in vivo models is necessary to determine the long-term effects of BPA-induced mitochondrial stress, especially in the context of tumor growth. These results highlight the need for additional research to elucidate BPA’s role in CC and assess its possible consequences for public health, given the widespread human exposure to BPA.

## Supplementary information

Below is the link to the electronic supplementary material.

ESM 1 Overall survival analysis of the top 10 hub genes (PNG 1.47 MB)

ESM 1 High Resolution Image (TIF 3.76 MB)

## Data Availability

No datasets were generated or analyzed during the current study.

## Abbreviations

ATP: Adenosine triphosphate
BCL2: B-cell lymphoma 2
BIGs: BPA interactive genes
BPA: Bisphenol A
BSA: Bovine serum albumin
Ca^2+^: Calcium
CAT: Catalase
CC: Cervical cancer
COX-2: Cyclooxygenase 2
CTD: Comparative Toxicogenomics databases
DMEM: Dulbecco’s modified Eagle’s medium
DRP1: Dynamin-related protein 1
EDCs: Endocrine-disrupting chemicals
ERα and β: Estrogen receptors α and β
GSH: Glutathione
LAXs: Leica Application Suite X software
LPR: Lipid peroxidation rate
miRNA: MicroRNA
mtDNA: Mitochondrial DNA
MM: Mitochondrial mass
MMP: Mitochondrial membrane potential
NAO: Nonyl Acridine Orange
OCR: Oxygen consumption rate
PBS: Phosphate-buffered saline
PPIN: Protein‒protein interaction networks
RMSD: Root mean square deviation
RMSF: Root mean square fluctuation
ROS: Reactive oxygen species
RPMI: Roswell Park Memorial Institute
SD: Standard deviation
SOD: Superoxide dismutase
SORA: Sorafenib
TACCO: Transcriptome Alterations in the CanCer Omnibus
TCGA: The Cancer Genome Atlas
